# Terminal trajectory of HbA_1c_ for 10 years supports the HbA_1c_ paradox: a longitudinal study using Health and Retirement Study data

**DOI:** 10.3389/fendo.2024.1383516

**Published:** 2024-04-22

**Authors:** Zeyi Zhang, Longshan Yang, Heng Cao

**Affiliations:** ^1^ Department of Surgical Intensive Care Unit, Shandong Provincial Hospital Affiliated to Shandong First Medical University, Jinan, China; ^2^ Department of General Surgery, Qilu Hospital of Shandong University, Jinan, China

**Keywords:** glycated hemoglobin (HbA_1c_), mortality, trajectory, terminal decline, time-varying

## Abstract

**Objectives:**

We aimed to assess the potential time-varying associations between HbA_1c_ and mortality, as well as the terminal trajectory of HbA_1c_ in the elderly to reveal the underlying mechanisms.

**Design:**

The design is a longitudinal study using data from the Health and Retirement Study.

**Setting and participants:**

Data were from the Health and Retirement Study. A total of 10,408 participants aged ≥50 years with available HbA_1c_ measurements at baseline (2006/2008) were included.

**Methods:**

Longitudinal HbA_1c_ measured at 2010/2012 and 2014/2016 were collected. HbA_1c_ values measured three times for their associations with all-cause mortality were assessed using Cox regression and restricted cubic splines. HbA_1c_ terminal trajectories over 10 years before death were analyzed using linear mixed-effect models with a backward time scale.

**Results:**

Women constitute 59.6% of the participants with a mean age of 69 years, with 3,070 decedents during the follow-up (8.9 years). The mortality rate during follow-up was 29.5%. Increased mortality risk became insignificant for the highest quartile of HbA_1c_ compared to the third quartile (aHR 1.148, 1.302, and 1.069 for a follow-up of 8.9, 6.5, and 3.2 years, respectively) with a shorter follow-up, while it became higher for the lowest quartile of HbA_1c_ (aHR 0.986, 1.068, and 1.439 for a follow-up of 8.9, 6.5, and 3.2 years, respectively). Accordingly, for both decedents with and without diabetes, an initial increase in HbA_1c_ was followed by an accelerating terminal decline starting 5–6 years before death.

**Conclusions and implications:**

The time-varying association between HbA_1c_ and mortality mapped to the terminal trajectory in HbA_1c_. High and low HbA_1c_ may have different clinical relationships with mortality. The HbA_1c_ paradox may be partially explained by reverse causation, namely, early manifestation of death.

## Highlights

This study found a time-varying association of HbA_1c_ and mortality as death approaches, indicating an initial rise and then a decline in the terminal trajectory of HbA_1c_. The HbA_1c_ paradox may be explained by reverse causation.

## Introduction

The average plasma glucose level for the 2 to 3 months prior was reflected by glycated hemoglobin (HbA_1c_) (1), which increases with age (2–4). It is acknowledged that a high HbA_1c_ level is an independent indicator of mortality among middle-aged and older adults with and without diabetes (5, 6). Nevertheless, studies demonstrating the detrimental effects of low HbA_1c_ and the protective benefit of high HbA_1c_ have emerged over the past few decades, which is counterintuitive and known as the HbA_1c_ paradox. This inconsistency regarding the association between HbA_1c_ and mortality was noteworthy among older adults (7–9). According to a recent large-scale observational study conducted in primary care in the UK, this inconsistency may be interpreted in terms of possible varying magnitudes of the association between HbA_1c_ and mortality over time (8). Indeed, the effect of HbA_1c_ on mortality may vary across follow-up (10), which is seldom considered in prior studies.

Several researchers have highlighted the potential of reverse causation for explaining the varied effects of HbA_1c_ on mortality, or sometimes known as the HbA_1c_ paradox. This suggests that rather than being randomly associated with long-term mortality, low HbA_1c_ may instead be associated with poor health in older adults (11, 12), which could be an early sign of mortality. However, such hypothesis has not been examined using formal analyses. Terminal trajectories with a backward time scale (13) can be used to characterize the exact changes of HbA_1c_ in the years immediately before death, and therefore can be applied to examine the early manifestations of death. Furthermore, several studies that have modeled biomarker terminal trajectories as time to death in older adults have identified a terminal decline for blood pressure (BP) (14–16) and total cholesterol (TC) (17), indicating that low BP or TC is a proxy of mortality. Nevertheless, the terminal trajectory of HbA_1c_ with proximity to death has not been assessed. Addressing such an issue will not only shed light on the understanding of the time-varying association of HbA_1c_ with mortality from a clinical perspective, but also inform risk assessment and treatment decision-making.

Therefore, the aims of this study were (1) to investigate the associations between HbA_1c_ measured at multiple times and mortality to assess the potential time-varying association, and (2) to estimate the terminal HbA_1c_ trajectories as death approached and compare them with the HbA_1c_ trajectory for survivors over a 10-year period in a middle-aged and older population with and without diabetes.

## Materials and methods

### Study population

This study used data from the Health and Retirement Study (HRS). HRS is a nationally representative longitudinal survey of noninstitutionalized older adults aged ≥50 years in the United States (18). Biomarker measures including HbA_1c_ are examined during the enhanced face-to-face interview, with a randomized half of the sample first measured in 2006 and the other half measured in 2008. Each group then had follow-up exams every 4 years following the initial exam. For this study, we used data covering 2006 over 2016. The study population (*n* = 10,852) consisted of respondents who satisfied the following criteria: (1) completed the interview in 2006 or 2008 (i.e., baseline); (2) had baseline HbA_1c_ measures; and (3) had reported their history of diabetes (yes/no) at baseline. We further excluded participants who had missing data for any of the baseline covariates (*n* = 444). A total of 23,273 interviews of 10,408 participants were eligible (mean, 2.2 interviews/person) (**Figure S1**). Of those with baseline HbA_1c_ measure, 7,286 (70.0%) participated during the second wave (2010/2012) and 5,801 (55.7%) participated during the third wave (2014/2016). Having baseline diabetes was defined as individuals who answered “yes” to the question “Has a doctor ever told you that you have diabetes or high blood sugar?” or had baseline HbA_1c_ ≥6.5% (19). In HRS, proxy interview rates across years (i.e., 2006–2016) ranged from 4.5% to 6.8%. All participants or their proxy respondents have provided written informed consent.

### HbA_1c_ measurement (2006/2008, 2010/2012, 2014/2016)

We used three waves of HbA_1c_ data collected at interviews of 2006/2008, 2010/2012, and 2014/2016 for analysis. In the HRS, HbA_1c_ was measured by dried blood spots (DBSs), which are highly correlated with the whole blood (*r* = 0.956) (20). Because the biomarker values based on DBS vary across assays and laboratories, the HRS adjusted DBS values to levels consistent with the National Health and Nutrition Examination Survey (NHANES) (21). We used the NHANES-equivalent assays for analysis, which are recommended by HRS and used in previous studies.

### Mortality

Deaths from any cause through 2018 and the date of death (year) were determined by exit interviews with proxy respondents as well as the National Death Index data linked to respondents. For this study, included participants who died from 2006 to 2018 are identified as decedents, and those who survived 2018 were identified as survivors.

### Covariates

Information on covariates were obtained from the baseline interview, including sociodemographic variables (sex, age, race, education, and marital status), lifestyle variables [physical activity, smoking, drinking, and body mass index (BMI)], and health conditions (diabetes, hypertension, heart diseases, and multimorbidity score). Details were presented in the **Supplementary Materials** (**eMethod 1**).

### Statistical analyses

Baseline characteristics of participants by survival status at the end of follow-up were described. Comparisons between decedents and survivors were performed using *t*-test, Kruskal–Wallis test, or chi-square test.

Three Cox proportional regression models were used to examine the time-varying association between HbA_1c_ and mortality risk. HbA_1c_ values measured in 2006/2008, 2010/2012, and 2014/2016 were, respectively, the independent variables in the three models. Follow-up was from HbA_1c_ measurements until death or December 2018. As a result, the time-varying connection between HbA_1c_ and mortality can be examined under various follow-up durations (as shown in **Figure S2**). In particular, the quartiles of the baseline HbA_1c_ test were used to classify the HbA_1c_ values obtained in the three waves. We reported the hazard ratios (HRs) and 95% confidence intervals (CIs) for different HbA_1c_ categories in the three models respectively. Based on earlier research suggesting that 5.6%–6.5% may be the ideal ranges for overall survival, we selected the third quartile as our reference (22). Proportional hazards assumption was tested by the Schoenfeld residuals trend test (all *p* > 0.333). Analyses were adjusted for sociodemographic variables (model 1), additionally for lifestyle variables (model 2), and then for health condition variables (model 3). Restricted cubic splines (RCS) with four knots (selected based on model R^2^) were applied to visualize the nonlinear association between continuous HbA_1c_ and mortality across different exposure times.

We estimated the terminal trajectory of HbA_1c_ using linear mixed models. Models were fitted with HbA_1c_ as the dependent variable, and survival status, time terms, and their interactions as independent variables. As for the time terms, we adopted a backward time scale spanning to 10 years before death or end of follow-up. The random effects for the intercept and time in the linear mixed models allowed for differences in HbA_1c_ at the intercept (time 0) and change in HbA_1c_ over time. Models were adjusted for age at death and covariates mentioned above. For better comprehension, continuous variables were centered around the baseline mean. Because of the non-normal distribution, the HbA_1c_ values were converted logarithmically, and the coefficients can be understood as percentage differences in means.

We performed subgroup analysis by diabetes status at baseline in all of the above analyses. For sensitivity analyses, we additionally adjusted for self-reported medication use relating to hypertension, diabetes, and heart diseases at baseline. Moreover, to control for hemoglobinopathy, we excluded participants with HbA_1c_ < 4.5% (*n* = 99). We also excluded those who had a diagnosis of cancer at baseline (*n* = 1701). Finally, we included participants who had missing covariates (*n* = 444) and re-analyzed by imputing the missing values. Statistical significance was set to two-sided *p* < 0.05. STATA version 14 and R version 4.1.2 were used for analyses (23, 24). Details about statistical analyses are provided in the **Supplementary Materials** (**eMethod 2**).

## Results

Of the 10,408 included participants, 3,070 were identified as decedents while the remaining 7,338 were identified as survivors with a 5-year follow-up mortality rate of 13.3% and 29.5%, respectively, during the full follow-up. **Table 1** displays the baseline characteristics by survival status. Decedents were older at baseline (76.3 vs. 65.9 years) and time 0 (81.2 vs. 74.1 years), and had a higher proportion of men (47.0% vs. 37.6%) and self-reported history of diabetes at baseline [27.2% (*n* = 836) vs. 17.7% (*n* = 1,301)]. The median level of HbA_1c_ at baseline was 5.8% in decedents and 5.6% in survivors. There were also differences between decedents and survivors in other sociodemographic, lifestyle, and health condition variables. Respondents who were excluded from the study were older, had more comorbidities, and had higher HbA_1c_ (**Table S1**).

**Table 1:** Baseline characteristics (2006/2008) of participants by survival status at the end of the follow-up (2018).

Characteristics	Total	Survival status at 2018 (time 0)		
		Decedents (*n* = 3,070)	Survivors (*n* = 7,338)	*p*-value
**Age, years**	69.0 ± 10.4	76.3 ± 9.4	65.9 ± 9.2	<0.001
**Female**	6,206 (59.6)	1,626 (53.0)	4,580 (62.4)	<0.001
**Race**				0.003
White	8,594 (82.6)	2,554 (83.2)	6,040 (82.3)	
Black	1,328 (12.8)	406 (13.2)	922 (12.6)	
Other	486 (4.7)	110 (3.6)	376 (5.1)	
**Education**				<0.001
Below high school	2,279 (21.9)	895 (29.2)	1,384 (18.9)	
High school	3,559 (34.2)	1,097 (35.7)	2,462 (33.6)	
College and above	4,570 (43.9)	1,078 (35.1)	3,492 (47.6)	
**Marital status**				<0.001
Married	5,529 (53.1)	1,371 (44.7)	4,158 (56.7)	
Divorced	1,642 (15.8)	430 (14.0)	1,212 (16.5)	
Widowed	3,237 (31.1)	1,269 (41.3)	1,968 (26.8)	
**Ever smoke**	1,476 (14.2)	499 (16.3)	977 (13.3)	<0.001
**Ever drink**	5,428 (52.2)	1,364 (44.4)	4,064 (55.4)	<0.001
**Physical activity at recommended levels**	5,873 (56.4)	1,305 (42.5)	4,568 (62.3)	<0.001
**BMI, kg/m^2^**	33.2 (28.4–38.6)	32.2 (27.4–37.9)	33.5 (28.8–38.9)	<0.001
**Hypertension**	5,830 (56.4)	2,015 (66.0)	3,815 (52.3)	<0.001
**Diabetes**	2,137 (20.5)	836 (27.2)	1,301 (17.7)	<0.001
Heart diseases*	2,598 (25.0)	1,194 (38.9)	1,404 (19.1)	<0.001
**Multimorbidity score** ^†^	2.0 (1.0–3.0)	3.0 (2.0–4.0)	2.0 (1.0–3.0)	<0.001
**HbA_1c_ at baseline, %**	5.7 (5.3–6.1)	5.8 (5.4–6.3)	5.6 (5.2–6.0)	<0.001
**HbA_1c_ at 2010/2012, %**	5.7 (5.3–6.1)	5.8 (5.4–6.2)	5.6 (5.3–6.1)	<0.001
**HbA_1c_ at 2014/2016, %**	5.7 (5.4–6.3)	5.9 (5.4–6.4)	5.7 (5.4–6.2)	0.022
**Age at time 0, years** ^‡^	76.2 ± 9.9	81.2 ± 9.7	74.1 ± 9.2	<0.001

Values are mean ± SD, n (%), or median (interquartile range).

BMI, body mass index; HbA_1c_, glycated hemoglobin.

* Heart diseases included heart attack, coronary heart disease, angina, and congestive heart failure.

^†^ Multimorbidity score was created as the count of nine chronic diseases: hypertension, diabetes, heart diseases, lung diseases, stroke, cancer, psychiatric problems, dementia/Alzheimer's disease, and arthritis.

^‡^ Time 0 was 2018 for survivors and date of death for participants who died between baseline (2006/2008) and 2018.

### Time-to-event analyses

**Figure 1:**
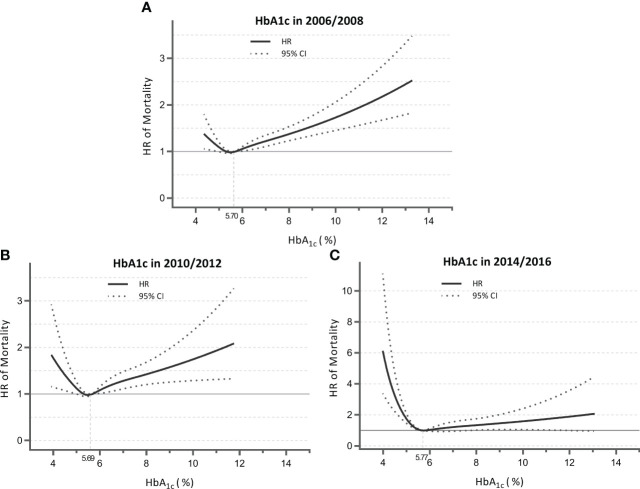
Nonlinear association of HbA_1c_ measured in 2006/2008 **(A)**, 2010/2012 **(B)**, and 2014/2016 **(C)** with mortality for total sample. Data were derived from the Cox models with restricted cubic splines. Analyses were adjusted for HbA_1c_; sex; age; race; marital status; education; physical activity; smoking; drinking; body mass index; history of hypertension, diabetes, and heart diseases; and multimorbidity score. HbA_1c_, glycated hemoglobin; HR, hazard ratio; CI, confidence interval.

We observed time-varying associations of HbA_1c_ with mortality across different follow-up durations (**Table 2**). For the Cox model with 2006/2008 HbA_1c_ measures as the independent variable (mean follow-up, 8.9 years), the highest quartile showed a significantly higher risk of mortality compared with the third quartile in all adjusted models (fully adjusted HR, 1.148; *p* = 0.013). This was also the case for the model of 2010/2012 (mean follow-up, 6.5 years; fully adjusted HR, 1.302; *p* = 0.001), but not for the model with a short follow-up (i.e., measures at 2014/2016; mean follow-up, 3.2 years; fully adjusted HR, 1.069; *p* = 0.597). Conversely, the lowest quartile of HbA_1c_ became significantly associated with a 43.9% higher mortality risk relative to the third quartile (*p* = 0.006) as follow-up was shorter (e.g., measures at 2014/2016). These temporal relationships between HbA_1c_ and mortality were visualized using RCS (**Figure 1**), showing a shift from “J-shape” to “L-shape” as the HbA_1c_ measurements get closer to death.

**Table 2:** Association between multiple measurements of HbA_1c_ and subsequent mortality.

Variables^†^	Model 1^*^		Model 2^*^		Model 3^*^	
	Hazard ratio (95% CI)	*p*-value	Hazard ratio (95% CI)	*p*-value	Hazard ratio (95% CI)	*p*-value
HbA_1c_ in 2006/2008^‡^ (mean follow-up 8.9 years)						
1st quartile	0.915 (0.821, 1.020)	0.108	0.911 (0.817, 1.016)	0.094	0.986 (0.883, 1.101)	0.803
2nd quartile	0.952 (0.857, 1.058)	0.360	0.963 (0.867, 1.070)	0.488	0.997 (0.897, 1.108)	0.952
3rd quartile	Ref.		Ref.		Ref.	
4th quartile	1.237 (1.117, 1.370)	<0.001	1.268 (1.144, 1.405)	<0.001	1.148 (1.029, 1.281)	0.013
HbA_1c_ in 2010/2012^§^ (mean follow-up 6.5 years)						
1st quartile	1.023 (0.875, 1.195)	0.777	1.022 (0.874, 1.194)	0.788	1.068 (0.912, 1.250)	0.414
2nd quartile	0.978 (0.842, 1.136)	0.772	0.993 (0.855 1.154)	0.931	1.034 (0.889, 1.202)	0.664
3rd quartile	Ref.		Ref.		Ref.	
4th quartile	1.417 (1.225, 1.639)	<0.001	1.421 (1.227, 1.644)	<0.001	1.302 (1.113, 1.523)	0.001
HbA_1c_ in 2014/2016^¶^ (mean follow-up 3.2 years)						
1st quartile	1.389 (1.075, 1.795)	0.012	1.422 (1.100, 1.838)	0.007	1.439 (1.112, 1.861)	0.006
2nd quartile	0.979 (0.746, 1.285)	0.880	0.973 (0.742, 1.277)	0.845	1.006 (0.766, 1.321)	0.976
3rd quartile	Ref.		Ref.		Ref.	
4th quartile	1.342 (1.072, 1.680)	0.010	1.288 (1.027, 1.616)	0.028	1.069 (0.836, 1.366)	0.597

HbA_1c_, glycated hemoglobin; CI, confidence interval.

^*^ Model 1 adjusted for sex, age, race, marital status, and education; Model 2 additionally adjusted for physical activity, smoking, drinking, and BMI; Model 3 additionally adjusted for history of hypertension, diabetes and heart diseases, and multimorbidity score.

^†^ HbA_1c_ was categorized according to the quartiles of HbA_1c_ measurements at baseline, i.e., 1st quartile (HbA_1c_ ≤ 5.34%), 2nd quartile (5.34% < HbA_1c_ ≤5.69%), 3rd quartile (5.69% < HbA_1c_ ≤ 6.14%), and 4th quartile (HbA_1c_ > 6.14%). We treated the third quartile (5.69% < HbA_1c_ ≤ 6.14%) as the reference in all the three Cox models.

^‡^ For HbA_1c_ measures at 2006/2008, there was a mean follow-up of 8.9 years (SD 2.5), with 92,188 person-years and deaths/total of 3,070/10,408.

^§^ For HbA_1c_ measures at 2010/2012, there was a mean follow-up of 6.5 years (SD 1.8), with 47,622 person-years and deaths/total of 1,455/7,286.

^¶^ For HbA_1c_ measures at 2014/2016, there was a mean follow-up of 3.2 years (SD 1.0), with 18,345 person-years and deaths/total of 520/5,801.

When stratified by diabetes status reported at baseline (**Table S2**; **Figure S3**), participants without diabetes showed that the highest quartile of HbA_1c_ was consistently associated with higher mortality risk compared with the third quartile across the three measurements. For participants with diabetes, there was a more significant increase in mortality risk for the lowest quartile of HbA_1c_ as follow-up was shorter.

### Terminal HbA_1c_ trajectories over 10 years

**Figure 2:**
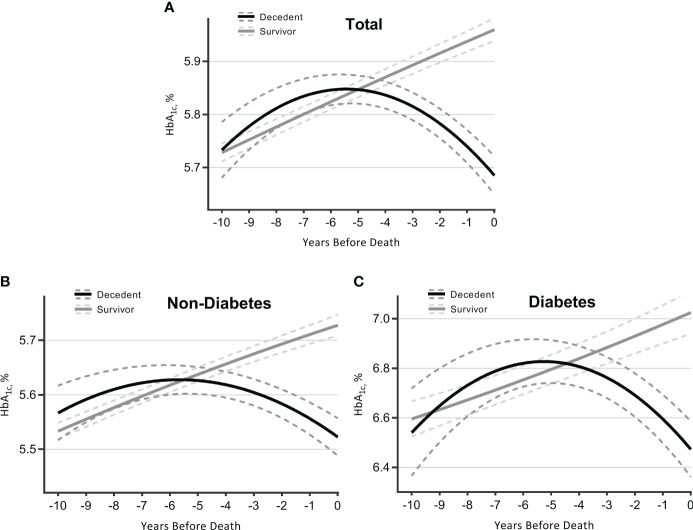
Trajectories of HbA_1c_ over 10 years before death (decedents, *n* = 3,070) or end of follow-up (survivors, *n* = 7,338). Estimated mean values were from linear mixed-effect models for total sample **(A)**, participants without diabetes **(B)**, and those with diabetes **(C)**. Analyses were adjusted for sex, age at time 0, race, marital status, education, smoking, drinking, body mass index, physical activity, hypertension, diabetes [not for figure **(B)** or **(C)**], heart diseases, multimorbidity score, survival status, time terms (time and time 2), and interactions of survival status and time terms. HbA_1c_, glycated hemoglobin.

The clinical reasons underlying the time-varying link between HbA_1c_ and mortality were further suggested by the terminal HbA_1c_ trajectories for survivors and deceased individuals. As indicated (**Figure 2A**), survivors experienced a consistent increase in HbA_1c_. In contrast, decedents initially experienced a higher level of and a steeper rise in HbA_1c_, peaking in years 5–6, and then declined more quickly as they approached death. An intersection point was captured at year 5, whereby survivors had a higher HbA_1c_ level than decedents. **Table 3** summarizes the intercept and slope differences of HbA_1c_ trajectories across survival status. There were significant interactions of survival status with linear time (*p* < 0.001) and quadratic time (*p* < 0.001), indicating an intensified decline in HbA_1c_ for decedents compared with survivors (**Table 3**).

**Table 3:** Coefficient estimates of HbA_1c_ trajectories for decedents and survivors for 10 years from linear mixed-effect models.

Variables	Total sample		Non-diabetes		Diabetes	
	Coefficient estimates (95% CI)	*p*-value	Coefficient estimates (95% CI)	*p*-value	Coefficient estimates (95% CI)	*p*-value
**Survival status^†^ (ref. survivors)**	−0.0205 (−0.0248, −0.0171)	<0.001	−0.0158 (−0.0190, −0.0126)	<0.001	−0.0355 (−0.0451, −0.0258)	<0.001
**Time^‡^**	0.0016 (0.0010, 0.0022)	<0.001	0.0013 (0.0007, 0.0019)	<0.001	0.0030 (0.0009, 0.0052)	0.006
**Time^2^**	−0.0000 (−0.0001, 0.0000)	0.658	−0.0000 (−0.0000, 0.0000)	0.407	−0.0000 (−0.0002, 0.0002)	0.778
**Survival status × Time**	−0.0061 (−0.0075, −0.0047)	<0.001	−0.0043 (−0.0055, −0.0028)	<0.001	−0.0118 (−0.0159, −0.0077)	<0.001
**Survival status × Time^2^**	−0.0004 (−0.0005, −0.0003)	<0.001	−0.0002 (−0.0004, −0.0001)	0.001	−0.0009 (−0.0013, −0.0004)	<0.001
**Intercept**	0.7651 (0.7615, 0.7686)	<0.001	0.7609 (0.7576, 0.7641)	<0.001	0.8672 (0.8564, 0.8781)	<0.001

Data presented in the table were derived from the linear mixed-effect models using the log of HbA_1c_ values. Models were adjusted for sex, age, race, marital status, education, physical activity, smoking, drinking, body mass index, history of hypertension, diabetes and heart diseases, and multimorbidity score.

CI, confidence interval.

^†^ Survival status at 2018, including decedents (n = 3,070) and survivors (n = 7,338).

^‡^ Time was the years before death or end of follow-up. Time 0 was 2018 for survivors and date of death for participants who died between baseline (2006/2008) and 2018.

Participants were further stratified by diabetes status collected at baseline. Those without diabetes followed a similar pattern of HbA_1c_ trajectories as observed in the total sample, although the overall HbA_1c_ levels were lower and changes were gentler (**Figure 2B**; **Table 3**). As for participants with diabetes (**Figure 2C**; **Table 3**), decedents showed a more remarkable upward trend than survivors until years 5–6, with a later intersection (at year 4.5) and a subsequently wider negative gap (i.e., decedents had lower HbA_1c_ levels) (**Table S3**).

### Sensitivity analyses

Sensitivity analyses as described above did not alter the results (**Tables S4**-**S10**).

## Discussion

This study shows that low HbA_1c_ is associated with higher mortality within short follow-up periods, while high HbA_1c_ is associated with higher mortality in long follow-up periods, indicating a change from a “J-shaped” to an “L-shaped” association between HbA_1c_ level and mortality as death approached. Accordingly, HbA_1c_ trajectory first rose and then terminally declined at an accelerated rate as death approached. Participants who had and those who did not have diabetes at baseline followed a similar pattern. These findings revealed that the effect of HbA_1c_ on mortality that varied across follow-up in older adults may be a reflection of terminal HbA_1c_ trajectory. As a result, there may be differences in the clinical and biological significance of high and low HbA_1c_ levels with respect to mortality risk. Reverse causality provides some explanation for the HbA_1c_ paradox.

### Comparisons with previous studies

Recently, the paradoxically protective effects of higher glycemia have been extensively investigated, with studies showing that HbA_1c_ <5.0% and ≥6.5% would increase 30-day, 90-day, and 1-year mortality for critically ill patients, as well as an approximate U-shape association between HbA_1c_ and the risk of mortality being recognized (25–28). As for long-term effects, similar results—subjects with HbA_1c_ level <6% and ≥10% were more likely to have in-hospitality mortality—were found (29). However, prior studies on the HbA_1c_ paradox have largely focused on mean HRs over the follow-up period, potentially ignoring the varied effects for the temporal pattern of exposure. A meta-analysis found that both high and low HbA_1c_ were risk factors for mortality, with duration of follow-up being one of the sources of heterogeneity (30). Accounting for this, Laiteerapong et al. reported that longer period of exposure to high HbA_1c_ (>8.0%) among adults with diabetes was linked to higher mortality risk (31). Comparably, a recent nationally representative study conducted in the United States showed that a low HbA_1c_ was associated with an elevated risk of all-cause death at 5 and 10 years of follow-up, respectively, of 30% and 12% (32). Although they involve different populations and HbA_1c_ levels, these findings were in parallel to our results, in which increased mortality risk became lower for high HbA_1c_, while it became higher for low HbA_1c_ when follow-up was shorter (i.e., closer to death). Such results highlight the necessity of taking into account differences in magnitude when evaluating the effect of HbA_1c_ on mortality. In particular, when stratified by diabetes status, we observed that increased mortality risk was consistently correlated to high HbA_1c_ for participants without diabetes, while it was more significantly correlated to low HbA_1c_ for participants with diabetes. Therefore, while strict glycemic control may not always assist those with diabetes, maintaining a healthy HbA_1c_ level over time may be crucial for those without the disease (33).

A growing number of studies argue that the observed increased mortality risk associated with low HbA_1c_ or intensive glucose therapy may not be a casual effect of low HbA_1c_ (32, 34); an epidemiological investigation using the United States national database also supported the idea that low HbA_1c_ level was a proxy of end stage of life after adjusting for an extensive set of potential confounders with flexible modeling (8). However, the above studies did not conduct a formal analysis of terminal HbA_1c_ trajectory in decedents. Our study found an accelerated terminal decline in HbA_1c_ over 5–6 years prior to death. Comparing survivors and decedents revealed that this terminal decline in HbA_1c_ might not be the result of aging. Furthermore, HbA_1c_-lowering treatment also did not seem to explain such a decline since participants without diabetes also experienced a terminal decline. Therefore, our results expanded earlier studies on terminal trajectories of cardiometabolic indicators (14, 15, 17) and provide straightforward evidence for the hypothesis that low HbA_1c_ is a sign of mortality. Although the exact mechanisms causing the observed terminal HbA_1c_ fall are unknown, they may have to do with failing organs, malnourishment, and unfavorable profiles of components associated with red blood cells, among others (11, 35). Indeed, the relationship between low HbA_1c_ and mortality was lessened in our study’s sensitivity analyses by removing people with an HbA_1c_ of less than 4.5% (probable hemoglobinopathy).

It is interesting to note that the decedents’ trajectory showed an early rise in HbA_1c_. Some post-trial analyses have presented the HbA_1c_ “legacy effect”; that is, exposure to adverse glucose control had a prolonged influence on the risk of future mortality (36). Several longitudinal studies have shown the adverse effects of poor glycemic control on major cardiovascular events and immune function related to COVID-19 over the years (37, 38). These data indicated that high HbA_1c_ level may be a predictor for the long-term mortality risk. Furthermore, the initially rising and subsequently falling HbA_1c_ trajectory is exactly mapped to the time-varying associations between HbA_1c_ and mortality across follow-up, offering a mechanism explanation for the time-varying association. In fact, our conclusion is in line with several clinical mechanism studies that show that early glycemic intervention targeting glucose sodium-cotransporter-2 inhibitors can improve myocardial function (39–41). Conjunctively, we supposed that high HbA_1c_ may be a long-term predictor of death, whereas low HbA_1c_ is more likely a reverse causation, namely, an early manifestation of mortality, for this middle-aged and older adult population.

### Meaning of findings

This study will be beneficial for the understanding of physiological change before death and the identification of risk populations. Results highlighted the importance of long-term HbA_1c_ monitoring as early as possible in light of the probability of notable alterations occurring 10 years before to mortality (42, 43). In a clinical setting, effective glycemic treatment can be introduced early to patients with increased HbA_1c_ (44, 45). However, an intensified glycemic control may not be appropriate for those who are at the end of life, supporting recent recommendations that the glycemic management of older adults with diabetes should be individualized depending on the patient’s life expectancy and overall health status (46, 47). Moreover, the identification of turning point at 5 years before death could have implications for the less glycemic targets in the context of life span. This study also supported the rationale of “reverse epidemiology/reverse causation” (48); that is, patients with a lower HbA_1c_ were generally closer to death, which may partly explain the paradoxical effects of HbA_1c_ in previous studies.

### Strengths and limitations

This study allowed for additional insight into the processes behind the time-varying effects of HbA_1c_ on mortality by combining retrospective terminal trajectory studies with prospective time-to-event analysis. Moreover, to our knowledge, this study provides the first evidence of the terminal trajectory in HbA_1c_ using a backward time scale for 10 years before death. Another advantage is the application of a nationally representative sample of middle-aged and older adults drawn from the HRS panel study. However, some limitations should be considered. Firstly, we are unable to examine the HbA_1c_ trajectory among specific causes of death because of a lack of information on cause-specific mortality. In addition, it remains unclear to what extent the diabetes phenotypes, treatments, and disease duration impact the HbA_1c_ terminal trajectory, although we managed to control the usage of medication in sensitivity analyses. There was evidence that patients with a long duration of diabetes were more inclined to have poor outcomes when exposed to low HbA_1c_ than those with a short duration (49). Therefore, future studies are expected to validate the present findings accounting for these factors. Moreover, we cannot completely rule out misclassification, nor did we take time-varying confounders into account. Information reported by agents at the time of data collection was incorporated into HRS, and this study may be exposed to additional unmeasured confounders. Moreover, we adopted the logarithmic transformation of HbA_1c_ data as the analytical variable in our study, which may have limited how our findings could be interpreted and directly compared to those of other investigations. Furthermore, contrary to what previous studies have found, our study did not identify a steeper decline in HbA_1c_ during the last 2 years before death (14, 17). This may arise from the relatively long measurement interval. Nevertheless, obtaining frequent HbA_1c_ in representative populations over 10 years before death can be challenging, and the present data with repeated measurements are qualified for analysis.

### Conclusions and implications

This analysis showed time-varying associations between HbA_1c_ and mortality across different durations of follow-up, shifting from “J-shaped” to “L-shaped” as death approached. Such associations are exactly mapped to the HbA_1c_ terminal trajectory showing an early increase followed by an accelerating terminal decline for 5–6 years before death in HbA_1c_, which was not observed in survivors. These results suggested that there might be differences in the clinical significance of high and low HbA_1c_ with regard to mortality: high HbA_1c_ may be a long-term predictor of death, while low HbA_1c_ may be an early manifestation of death. Thus, the HbA_1c_ conundrum in earlier research may be partially explained by reverse causation. Our findings underscore the necessity of long-term HbA_1c_ monitoring as early as possible. Additionally, effective glycemic control should be introduced early but may not be appropriate when patients are at the end of life.

## Data availability statement

The datasets presented in this study can be found in online repositories. The names of the repository/repositories and accession number(s) can be found in the article/**Supplementary Material**.

## Ethics statement

The studies involving humans were approved by Behavioral Sciences Committee institutional review board at University of Michigan. The studies were conducted in accordance with the local legislation and institutional requirements. The participants provided their written informed consent to participate in this study.

## Author contributions

HC: Writing – review & editing, Validation, Supervision, Resources, Funding acquisition, Conceptualization. ZZ: Writing – original draft, Software, Methodology, Investigation, Conceptualization. LY: Writing – review & editing, Validation, Software, Methodology, Investigation, Formal analysis, Data curation.
