# Supplementary material for: Terminal trajectory of HbA1c for 10 years supports the HbA1c paradox: a longitudinal study using Health and Retirement Study data
Source: Front Endocrinol (Lausanne). 2024 Apr 22;15:1383516. doi: 10.3389/fendo.2024.1383516 (PMC11070457; doi:10.3389/fendo.2024.1383516)
Supplement: Supplementary file 1 [file Presentation_1.pdf]

Supplement

Terminal Trajectory of HbA1c for 10 Years Supports HbA1c Paradox: Longitudinal Study Using Health and Retirement Study

Zeyi Zhang[a] (M.D. Zhzeyi@outlook.com), Longshan Yang[b] (M.D. 907576964@qq.com), Heng Cao[*a] (M.D. caoheng_25@163.com)

[a] Department of Surgical Intensive Care Unit, Shandong Provincial Hospital Affiliated to Shandong First Medical University, #246 JingWu Road, Jinan 250021, China.

[b] Department of General Surgery, Qilu Hospital of Shandong University, Jinan 250012, PR China.

Supplement Legends:

Table S1 Characteristics of respondents excluded from the study.

Table S2 Association between multiple measurements of HbA_{1c} and subsequent mortality stratified by diabetes status.

Table S3 Estimated mean values of HbA_{1c} for decedents and survivors in each of the 10 years before death or end of follow-up.

Table S4 Coefficient estimates of HbA_{1c} trajectories for decedents and survivors for 10 years from linear mixed-effect models (additionally adjusted for medication use).

Table S5 Association between multiple measurements of HbA1c and subsequent mortality (additionally adjusted for medication use).

Table S6 Coefficient estimates of HbA_{1c} trajectories for decedents and survivors for 10 years from linear mixed-effect models (excluding participants with HbA_{1c} < 4.5%).

Table S7 Association between multiple measurements of HbA1c and subsequent mortality (excluding participants with HbA_{1c} < 4.5%).

Table S8 Coefficient estimates of HbA_{1c} trajectories for decedents and survivors for 10 years from linear mixed-effect models (excluding participants with cancer at baseline).

Table S9 Association between multiple measurements of HbA_{1c} and subsequent mortality (excluding participants with cancer at baseline).

Table S10 Association between multiple measurements of HbA_{1c} and subsequent mortality (using covariate-imputed dataset).

Figure S1 Flow chart of participants included in the study.

Figure S2 Example diagram of the time-to-event analyses of the association between HbA_{1c} and mortality.

Figure S3 Nonlinear association of HbA_{1c} measured in 2006/2008, 2010/2012, and 2014/2016 with mortality for participants without diabetes (a, b, c) and with diabetes (d, e, f).

Table S1 Characteristics of respondents excluded from the study

Characteristics	Included Respondents (n=10408)	Excluded Respondents* (n=456)	P value
**Age, years**	69.0±10.4	70.9±12.2	<0.001
**Female**	6206(59.6)	254(55.7)	0.097
**Race**			<0.001
White	8594(82.6)	345(75.8)	
Black	1328(12.8)	92(20.2)	
Other	486(4.7)	418(4.0)	
**Education**			<0.001
Below high school	2279(21.9)	147(34.0)	
High school	3559(34.2)	131(30.3)	
College and above	4570(43.9)	154(35.6)	
**Marital status**			0.002
married	5529(53.1)	204(44.7)	
divorced	1642(15.8)	87(19.1)	
widowed	3237(31.1)	165(36.2)	
**Ever smoke**	1476(14.2)	61(14.8)	0.722
**Ever drink**	5428(52.2)	182(41.1)	<0.001
**Physical activity at recommended levels**	5873(56.4)	93(20.4)	<0.001
**BMI, kg/m^2**	33.2(28.4-38.6)	32.4(28.4-37.1)	<0.001
**Hypertension**	5830(56.4)	319(70.6)	<0.001
**Diabetes**	2137(20.5)	169(38.6)	<0.001
**Heart diseases***	2598(25.0)	165(37.7)	<0.001
**Multimorbidity score**[†]	2.0(1.0-3.0)	3.0(2.0-4.0)	<0.001
**Hab1c at baseline, %**	5.7(5.3-6.1)	5.8(5.5-6.6)	<0.001
**Hab1c at 2010/2012, %**	5.7(5.3-6.1)	6.0(5.5-6.6)	<0.001
**Hab1c at 2014/2016, %**	5.7(5.4-6.3)	6.0(5.7-6.6)	0.022
**Age at time 0, years**[‡]	76.2±9.9	76.3±11.2	<0.001

Values are mean ± SD, n (%), or median (interquartile range).

BMI, body mass index; HbA1c, glycated hemoglobin

* Excluded Participants were those who had missing data on history of diabetes, covariates values at baseline.

[†] Heart diseases included heart attack, coronary heart disease, angina, and congestive heart failure.

[‡] Multimorbidity score was created as the count of nine chronic diseases: hypertension, diabetes, heart diseases, lung diseases, stroke, cancer, psychiatric problems, dementia/Alzheimer's Disease, and arthritis.

§ Time 0 was 2018 for survivors and date of death for participants who died between baseline (2006/2008) and 2018.

Table S2 Association between multiple measurements of HbA_{1c} and subsequent mortality stratified by diabetes status

Variables[†]	Model 1[*]		Model 2[*]		Model 3[*]	
	Hazard Ratio (95% CI)	*P* value	Hazard Ratio (95% CI)	*P* value	Hazard Ratio (95% CI)	*P* value
**Non-Diabetes**						
**HbA_{1c} in 2006/2008**						
1st quartile	0.929 (0.826, 1.045)	0.219	0.931 (0.828, 1.048)	0.236	0.973 (0.864, 1.096)	0.653
2nd quartile	0.961 (0.857, 1.078)	0.500	0.978 (0.872, 1.097)	0.705	0.985 (0.878, 1.105)	0.795
3rd quartile	Ref.		Ref.		Ref.	
4th quartile	1.058 (0.922, 1.213)	0.423	1.100 (0.958, 1.262)	0.176	1.132 (0.986, 1.299)	0.079
**HbA_{1c} in 2010/2012**						
1st quartile	1.042 (0.881, 1.233)	0.632	1.041 (0.880, 1.232)	0.640	1.053 (0.890, 1.247)	0.548
2nd quartile	1.007 (0.855 1.185)	0.935	1.024 (0.870 1.205)	0.776	1.051 (0.892, 1.237)	0.553
3rd quartile	Ref.		Ref.		Ref.	
4th quartile	1.329 (1.095, 1.613)	0.004	1.315 (1.083, 1.597)	0.006	1.359 (1.118, 1.652)	0.002
**HbA_{1c} in 2014/2016**						
1st quartile	1.395 (1.057, 1.841)	0.019	1.422 (1.077, 1.877)	0.013	1.417(1.073, 1.871)	0.014
2nd quartile	1.067 (0.801, 1.421)	0.660	1.059 (0.794, 1.411)	0.697	1.066 (0.800, 1.420)	0.664
3rd quartile	Ref.		Ref.		Ref.	
4th quartile	1.367 (1.036, 1.806)	0.027	1.362 (1.030, 1.800)	0.030	1.287 (0.972, 1.705)	0.079
**Diabetes**						
**HbA_{1c} in 2006/2008**						
1st quartile	1.213 (0.861, 1.708)	0.270	1.143 (0.811, 1.610)	0.445	1.106 (0.784, 1.560)	0.565
2nd quartile	1.099 (0.832, 1.453)	0.505	1.063 (0.804, 1.406)	0.666	1.048 (0.793, 1.386)	0.740
3rd quartile	Ref.		Ref.		Ref.	
4th quartile	1.216 (1.002, 1.476)	0.048	1.188 (0.978, 1.443)	0.077	1.189 (0.978, 1.446)	0.082
**HbA_{1c} in 2010/2012**						
1st quartile	1.582 (0.980, 2.554)	0.060	1.543(0.954, 2.496)	0.085	1.502 (0.929, 2.430)	0.097
2nd quartile	1.079 (0.709, 1.642)	0.723	1.033 (0.677, 1.576)	0.881	0.954 (0.624, 1.458)	0.827
3rd quartile	Ref.		Ref.		Ref.	
4th quartile	1.215 (0.921, 1.604)	0.168	1.210 (0.917, 1.597)	0.178	1.216 (0.921, 1.606)	0.168
**HbA_{1c} in 2014/2016**						

1st quartile	2.514 (1.262, 5.009)	0.009	2.453 (1.229, 4.894)	0.011	2.241 (1.112, 4.515)	0.024
2nd quartile	0.580 (0.199, 1.691)	0.319	0.556 (0.190, 1.626)	0.283	0.612 (0.207, 1.810)	0.375
3rd quartile	Ref.		Ref.		Ref.	
4th quartile	0.800 (0.507, 1.265)	0.340	0.737 (0.465, 1.169)	0.195	0.734 (0.461, 1.169)	0.193

HbA_{1c}, glycated hemoglobin; CI, confidence interval

* Model 1 adjusted for sex, age, race, marital status, and education; Model 2 additionally adjusted for physical activity, smoking, drinking, and BMI; Model 3 additionally adjusted for history of hypertension, diabetes and heart diseases, and multimorbidity score.

† HbA_{1c} were categorized according to the quartiles of HbA_{1c} measurements at baseline, i.e., 1st quartile ($HbA_{1c} \leq 5.34\%$), 2nd quartile ($5.34\% < HbA_{1c} \leq 5.69\%$), 3rd quartile ($5.69\% < HbA_{1c} \leq 6.14\%$), 4th quartile ($HbA_{1c} > 6.14\%$). We treated the third quartile ($5.69\% < HbA_{1c} \leq 6.14\%$) as the reference in all the Cox models.

Table S3 Estimated mean values of HbA1c for decedents and survivors in each of the 10 years before death or end of follow-up.

Years preceding T0*	Total sample			Non-diabetes			Diabetes		
	Mean values of HbA1c (%)		*P* value	Mean values of HbA1c (%)		*P* value	Mean values of HbA1c (%)		*P* value
	Decedents	Survivors		Decedents	Survivors		Decedents	Survivors	
-10	5.73	5.73	0.87	5.57	5.53	0.25	6.54	6.60	0.60
-9	5.78	5.75	0.28	5.59	5.56	0.10	6.65	6.63	0.86
-8	5.81	5.78	0.07	5.61	5.58	0.06	6.73	6.67	0.38
-7	5.83	5.80	0.05	5.62	5.60	0.13	6.79	6.71	0.21
-6	5.85	5.82	0.19	5.63	5.62	0.54	6.82	6.75	0.26
-5	5.85	5.85	0.99	5.63	5.64	0.49	6.83	6.80	0.60
-4	5.84	5.87	0.05	5.62	5.66	0.02	6.81	6.84	0.58
-3	5.81	5.89	<0.01	5.60	5.68	<0.01	6.76	6.88	0.03
-2	5.78	5.92	<0.01	5.58	5.69	<0.01	6.69	6.93	<0.01
-1	5.74	5.94	<0.01	5.56	5.71	<0.01	6.59	6.98	<0.01
0	5.69	5.96	<0.01	5.52	5.73	<0.01	6.47	7.02	<0.01
Mean change,% †	0.17			0.11			0.36		

HbA1c, glycated hemoglobin

* Years before death for decedents and end of follow-up for survivors. T0 is year of death for decedents and 2018 for survivors.

† Mean change from the peak year to time 0.

Table S4 Coefficient estimates of HbA1c trajectories for decedents and survivors for 10 years from linear mixed-effect models (additionally adjusted for medication use)

Variables	Total sample		Non-diabetes		Diabetes	
	Coefficient estimates (95% CI)	*P* value	Coefficient estimates (95% CI)	*P* value	Coefficient estimates (95% CI)	*P* value
Survival status† (ref. survivors)	-0.0214 (-0.0247, -0.0181)	<0.001	-0.0163 (-0.0195, -0.0130)	<0.001	-0.0377 (-0.0473, -0.0281)	<0.001
Time‡	0.0016 (0.0010, 0.0022)	<0.001	0.0013 (0.0007, 0.0019)	<0.001	0.0031 (0.0009, 0.0052)	0.005
Time ^{2}	-0.0000 (-0.0001, 0.0000)	0.681	-0.0000 (-0.0001, 0.0000)	0.407	-0.0000 (-0.0002, 0.0002)	0.763
Survival status × Time	-0.0062 (-0.0076, -0.0048)	<0.001	-0.0043 (-0.0056, -0.0029)	<0.001	-0.0117 (-0.0159, -0.0076)	<0.001
Survival status × Time ^{2}	-0.0004 (-0.0006, -0.0003)	<0.001	-0.0002 (-0.0004, -0.0001)	0.001	-0.0008 (-0.0013, -0.0004)	<0.001
Intercept	0.7636 (0.7601, 0.7671)	<0.001	0.7601 (0.7568, 0.7633)	<0.001	0.8312 (0.8193, 0.8432)	<0.001

Data presented in the table were derived from the linear mixed-effect models using the log of the HbA1c values. Models were adjusted for sex, age, race, marital status, education, physical activity, smoking, drinking, body mass index, history of hypertension, diabetes and heart diseases, multimorbidity score, and use of medication relating to hypertension, diabetes, and heart diseases.

CI, confidence interval.

† Survival status at 2018, including decedents (n=3070) and survivors (n=7338).

‡ Time was the years before death or end of follow-up. Time 0 was 2018 for survivors and date of death for participants who died between baseline (2006/2008) and 2018.

Table S5 Association between multiple measurements of HbA1c and subsequent mortality (additionally adjusted for medication use)

Variables[†]	Model 3*	
	Hazard Ratio (95% CI)	*P* value
**HbA1c in 2006/2008**		
1st quartile	0.980 (0.877, 1.094)	0.718
2nd quartile	0.993 (0.893, 1.104)	0.899
3rd quartile	Ref.	
4th quartile	1.128 (1.101, 1.259)	0.032
**HbA1c in 2010/2012**		
1st quartile	1.071 (0.915, 1.254)	0.392
2nd quartile	1.036 (0.891, 1.205)	0.642
3rd quartile	Ref.	
4th quartile	1.295 (1.105, 1.518)	0.001
**HbA1c in 2014/2016**		
1st quartile	1.434 (1.108, 1.855)	0.006
2nd quartile	1.009 (0.768 1.325)	0.949
3rd quartile	Ref.	
4th quartile	1.073 (0.838, 1.373)	0.557

HbA1c, glycated hemoglobin; CI, confidence interval

* Model 3 adjusted for sex, age, race, marital status, education, physical activity, smoking, drinking, body mass index, history of hypertension, diabetes and heart diseases, multimorbidity score, and use of medication relating to hypertension, diabetes, and heart diseases.

[†] HbA_{1c} were categorized according to the quartiles of HbA_{1c} measurements at baseline, i.e., 1st quartile ($HbA_{1c} \leq 5.34\%$), 2nd quartile ($5.34\% < HbA_{1c} \leq 5.69\%$), 3rd quartile ($5.69\% < HbA_{1c} \leq 6.14\%$), 4th quartile ($HbA_{1c} > 6.14\%$). We treated the third quartile ($5.69\% < HbA_{1c} \leq 6.14\%$) as the reference in all the Cox models.

Table S6 Coefficient estimates of HbA1c trajectories for decedents and survivors for 10 years from linear mixed-effect models (excluding participants with HbA1c < 4.5%)

Variables	Total sample		Non-diabetes		Diabetes	
	Coefficient estimates (95% CI)	*P* value	Coefficient estimates (95% CI)	*P* value	Coefficient estimates (95% CI)	*P* value
Survival status† (ref. survivors)	-0.0196 (-0.0229, -0.0163)	<0.001	-0.0150 (-0.0182, -0.0116)	<0.001	-0.0340 (-0.0437, -0.0244)	<0.001
Time‡	0.0015 (0.0009, 0.0022)	<0.001	0.0012 (0.0006, 0.0018)	<0.001	0.0030 (0.0008, 0.0051)	0.007
Time ^{2}	-0.0000 (-0.0001, 0.0000)	0.419	-0.0000 (-0.0001, 0.0000)	0.192	-0.0000 (-0.0002, 0.0002)	0.828
Survival status × Time	-0.0058 (-0.0072, -0.0044)	<0.001	-0.0038 (-0.0051, -0.0024)	<0.001	-0.0116 (-0.0157, -0.0075)	<0.001
Survival status × Time ^{2}	-0.0004 (-0.0005, -0.0002)	<0.001	-0.0002 (-0.0003, -0.0001)	0.005	-0.0009 (-0.0013, -0.0004)	<0.001
Intercept	0.7657 (0.7622, 0.7692)	<0.001	0.7616 (0.7583, 0.7648)	<0.001	0.8670 (0.8561, 0.8778)	<0.001

Data presented in the table were derived from the linear mixed-effect models using the log of the HbA1c values. Models were adjusted for sex, age, race, marital status, education, physical activity, smoking, drinking, body mass index, history of hypertension, diabetes and heart diseases, and multimorbidity score.

CI, confidence interval.

† Survival status at 2018, including decedents (n=3070) and survivors (n=7338).

‡ Time was the years before death or end of follow-up. Time 0 was 2018 for survivors and date of death for participants who died between baseline (2006/2008) and 2018.

Table S7 Association between multiple measurements of HbA1c and subsequent mortality (excluding participants with HbA1c < 4.5%)

**Variables**†	**Model 3***	
	**Hazard Ratio (95% CI)**	***P* value**
**HbA1c in 2006/2008**		
1st quartile	0.979 (0.877, 1.094)	0.713
2nd quartile	0.996 (0.896, 1.108)	0.948
3rd quartile	Ref.	
4th quartile	1.150 (1.031, 1.283)	0.012
**HbA1c in 2010/2012**		
1st quartile	1.048 (0.894, 1.228)	0.564
2nd quartile	1.035 (0.890, 1.203)	0.658
3rd quartile	Ref.	
4th quartile	1.302 (1.113, 1.524)	0.001
**HbA1c in 2014/2016**		
1st quartile	1.341 (1.031, 1.746)	0.029
2nd quartile	1.005(0.765, 1.320)	0.973
3rd quartile	Ref.	
4th quartile	1.073 (0.839, 1.372)	0.573

HbA1c, glycated hemoglobin; CI, confidence interval

* Model 3 adjusted for sex, age, race, marital status, education, physical activity, smoking, drinking, body mass index, history of hypertension, diabetes and heart diseases, and multimorbidity score.

† HbA_{1c} were categorized according to the quartiles of HbA_{1c} measurements at baseline, i.e., 1st quartile ($HbA_{1c} \leq 5.34\%$), 2nd quartile ($5.34\% < HbA_{1c} \leq 5.69\%$), 3rd quartile ($5.69\% < HbA_{1c} \leq 6.14\%$), 4th quartile ($HbA_{1c} > 6.14\%$). We treated the third quartile ($5.69\% < HbA_{1c} \leq 6.14\%$) as the reference in all the Cox models.

Table S8 Coefficient estimates of HbA1c trajectories for decedents and survivors for 10 years from linear mixed-effect models (excluding participants with cancer at baseline)

Variables	Total sample		Non-diabetes		Diabetes	
	Coefficient estimates (95% CI)	*P* value	Coefficient estimates (95% CI)	*P* value	Coefficient estimates (95% CI)	*P* value
Survival status† (ref. survivors)	-0.0204 (-0.0242, -0.0167)	<0.001	-0.0168 (-0.0205, -0.0132)	<0.001	-0.0325 (-0.0436, -0.0213)	<0.001
Time‡	0.0015 (0.0008, 0.0021)	<0.001	0.0012 (0.0006, 0.0019)	<0.001	0.0025 (0.0002, 0.0049)	0.036
$Time^2$	-0.0000 (-0.0001, 0.0000)	0.428	-0.0000 (-0.0001, 0.0000)	0.310	-0.0000 (-0.0002, 0.0002)	0.959
Survival status × Time	-0.0057 (-0.0073, -0.0041)	<0.001	-0.0045 (-0.0061, -0.0030)	<0.001	-0.0093 (-0.0141, -0.0045)	<0.001
Survival status × $Time^2$	-0.0004 (-0.0005, -0.0002)	<0.001	-0.0003 (-0.0004, -0.0001)	0.002	-0.0006 (-0.0011, -0.0001)	0.015
Intercept	0.7648 (0.7608, 0.7687)	<0.001	0.7606 (0.7569, 0.7643)	<0.001	0.8670 (0.8561, 0.8778)	<0.001

Data presented in the table were derived from the linear mixed-effect models using the log of the HbA1c values. Models were adjusted for sex, age, race, marital status, education, physical activity, smoking, drinking, body mass index, history of hypertension, diabetes and heart diseases, and multimorbidity score.

CI, confidence interval.

† Survival status at 2018, including decedents (n=3070) and survivors (n=7338).

‡ Time was the years before death or end of follow-up. Time 0 was 2018 for survivors and date of death for participants who died between baseline (2006/2008) and 2018.

Table S9 Association between multiple measurements of HbA1c and subsequent mortality (excluding participants with cancer at baseline)

Variables†	Model 3*	
	**Hazard Ratio (95% CI)**	***P* value**
**HbA1c in 2006/2008**		
1st quartile	0.970 (0.855, 1.101)	0.639
2nd quartile	0.978 (0.866, 1.104)	0.716
3rd quartile	Ref.	
4th quartile	1.143 (1.008, 1.296)	0.036
**HbA1c in 2010/2012**		
1st quartile	1.020 (0.851, 1.222)	0.831
2nd quartile	0.978 (0.822, 1.163)	0.802
3rd quartile	Ref.	
4th quartile	1.298 (1.085, 1.554)	0.004
**HbA1c in 2014/2016**		
1st quartile	1.562 (1.165, 2.094)	0.003
2nd quartile	0.998 (0.728, 1.367)	0.990
3rd quartile	Ref.	
4th quartile	1.107 (0.833, 1.472)	0.483

glycated hemoglobin; CI, confidence interval

* Model 3 adjusted for sex, age, race, marital status, education, physical activity, smoking, drinking, body mass index, history of hypertension, diabetes and heart diseases, and multimorbidity score.

† HbA_{1c} were categorized according to the quartiles of HbA_{1c} measurements at baseline, i.e., 1st quartile ($HbA_{1c} \leq 5.34\%$), 2nd quartile ($5.34\% < HbA_{1c} \leq 5.69\%$), 3rd quartile ($5.69\% < HbA_{1c} \leq 6.14\%$), 4th quartile ($HbA_{1c} > 6.14\%$). We treated the third quartile ($5.69\% < HbA_{1c} \leq 6.14\%$) as the reference in all the Cox models.

Table S10 Association between multiple measurements of HbA1c and subsequent mortality (using covariate-imputed dataset)

Variables†	Model 3*	
	Hazard Ratio (95% CI)	*P* value
**HbA1c in 2006/2008**		
1st quartile	1.008 (0.907, 1.121)	0.88
2nd quartile	1.006 (0.909, 1.114)	0.905
3rd quartile	Ref.	
4th quartile	1.148 (1.033, 1.274)	0.01
**HbA1c in 2010/2012**		
1st quartile	1.103 (0.950, 1.281)	0.198
2nd quartile	1.030 (0.882, 1.203)	0.707
3rd quartile	Ref.	
4th quartile	1.292 (1.107, 1.508)	0.001
**HbA1c in 2014/2016**		
1st quartile	1.413 (1.094, 1.825)	0.008
2nd quartile	1.000(0.771, 1.297)	0.999
3rd quartile	Ref.	
4th quartile	1.056 (0.833, 1.340)	0.659

glycated hemoglobin; CI, confidence interval

* Model 3 adjusted for sex, age, race, marital status, education, physical activity, smoking, drinking, body mass index, history of hypertension, diabetes and heart diseases, and multimorbidity score.

† HbA_{1c} were categorized according to the quartiles of HbA_{1c} measurements at baseline, i.e., 1st quartile ($HbA_{1c} \leq 5.34\%$), 2nd quartile ($5.34\% < HbA_{1c} \leq 5.69\%$), 3rd quartile ($5.69\% < HbA_{1c} \leq 6.14\%$), 4th quartile ($HbA_{1c} > 6.14\%$). We treated the third quartile ($5.69\% < HbA_{1c} \leq 6.14\%$) as the reference in all the Cox models.

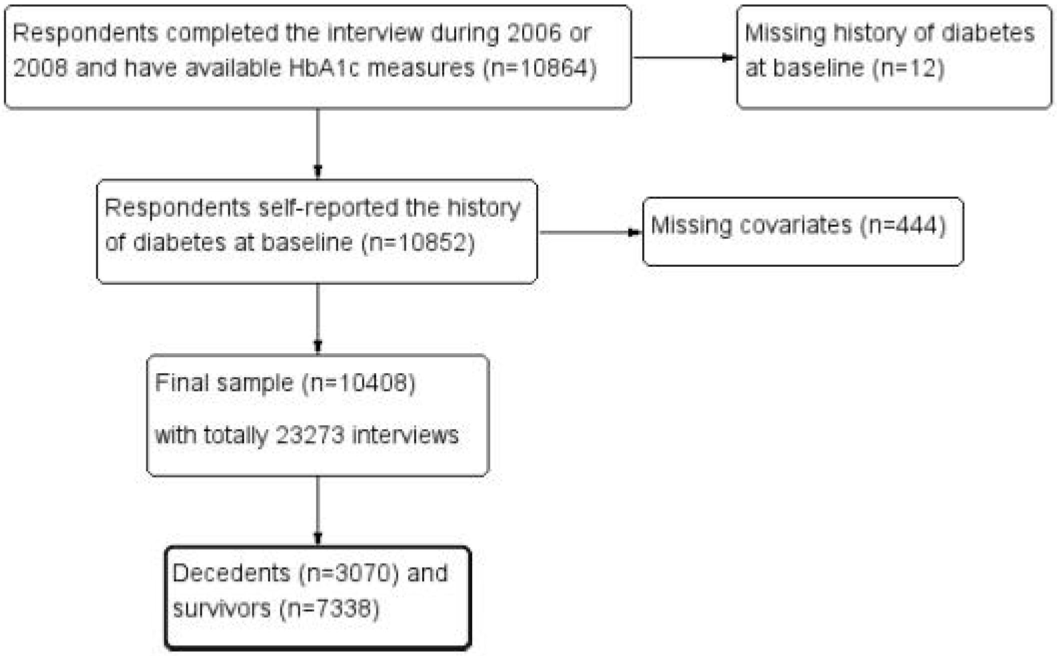

Figure S1 Flow chart of participants included in the study

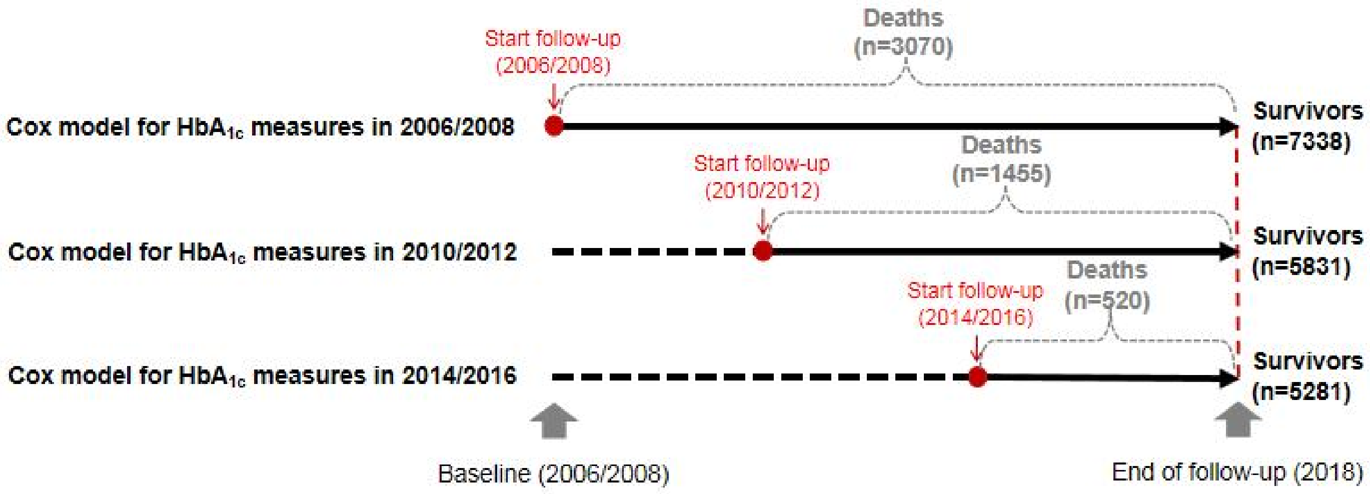

Figure S2 Example diagram of the time-to-event analyses of the association between HbA_{1c} and mortality

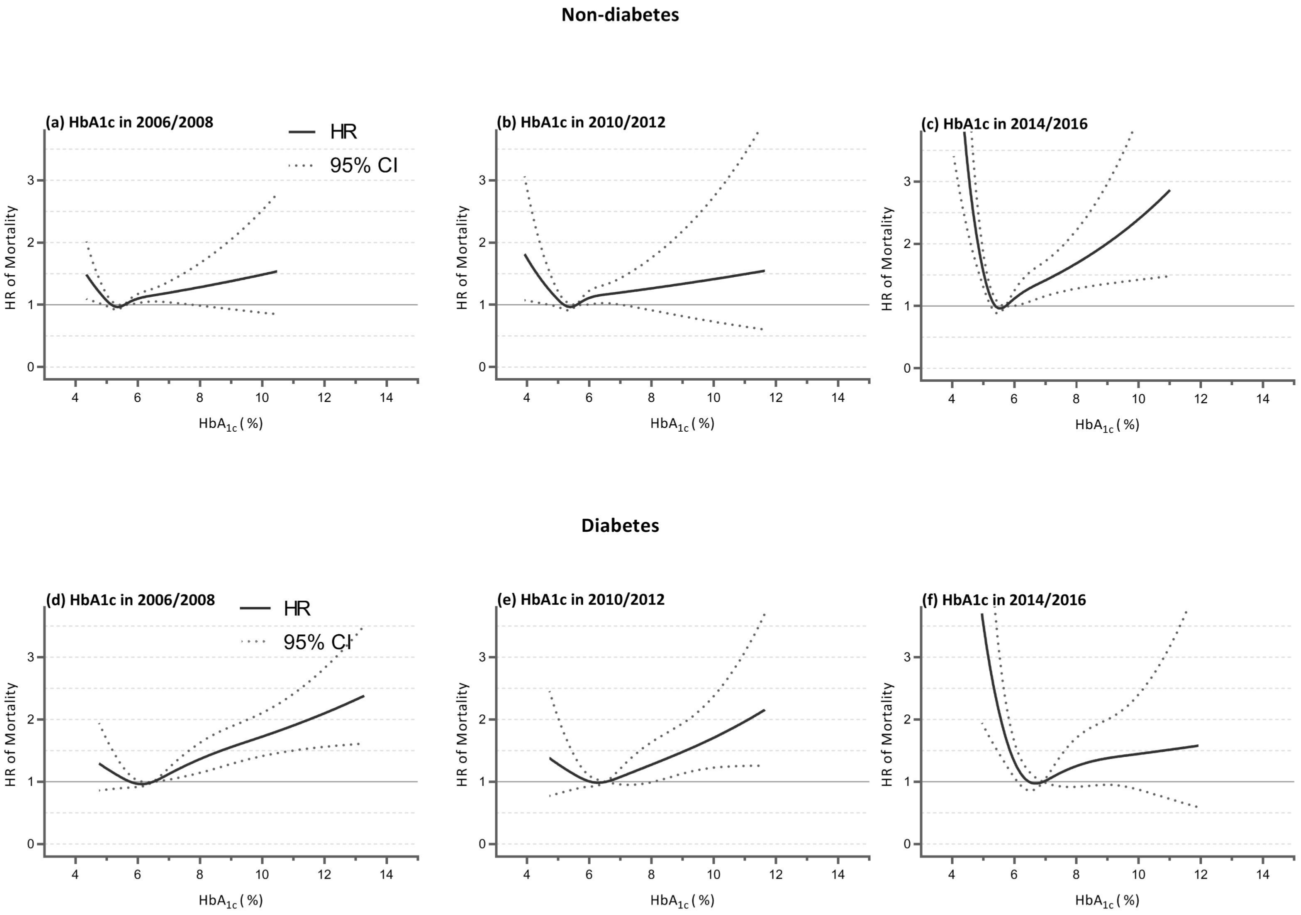

Figure S3 Nonlinear association of HbA1c measured in 2006/2008, 2010/2012, and 2014/2016 with mortality for participants without diabetes (a, b, c) and with diabetes (d, e, f). Data were derived from the Cox models with restricted cubic splines. Analyses were adjusted for HbA1c, sex, age, race, marital status, education, physical activity, smoke, drink, body mass index, history of hypertension and heart diseases, and multimorbidity score. HbA1c, glycated hemoglobin; HR, hazard ratio; CI, confidence interval

eMethod 1 Description of covariates.

Information on covariates were obtained from the baseline interview (i.e. 2006/2008) Sociodemographic variables included sex, age, race (white/black/other), education (below high school/high school/college and above), and marital status (married/divorced/widowed). Lifestyle variables included physical activity at recommended levels (yes/no), smoke (never/ever), and drink (never/ever) at baseline. Body mass index (BMI) was estimated using height and weight assessed at the baseline examination. Health conditions including self-reported history of diabetes, hypertension, and heart diseases (i.e. heart attack, coronary heart disease, angina, and congestive heart failure) at baseline. A multimorbidity score was created as the count of nine chronic diseases (hypertension, diabetes, heart diseases, lung diseases, stroke, cancer, psychiatric problems, dementia/Alzheimer's Disease, and arthritis). Covariates were selected according to their relations to HbA_{1c} and mortality in previous studies.

eMethod 2

Baseline characteristics of participants by survival status at the end of follow-up were described. Comparisons between decedents and survivors were performed using *t* test, Kruskal-Wallis test or Chi-square test. We estimated the terminal trajectory of HbA_{1c} using linear mixed models. Models were fitted with HbA_{1c} as the dependent variable, and survival status, time terms and their interactions as independent variables. As for the time terms, we adopted a backward time scale spanning to 10 years before death or end of follow-up, such that time 0 was 2018 for survivors and date of death for participants who died between baseline and 2018. To account for the nonlinear form of trajectories, quadratic and cubic polynomial time terms and their interactions with survival status were tested (cubic term was discarded due to non-significance for parsimonious model). The random effects for the intercept and time in the linear mixed models allowed for differences in HbA_{1c} at the intercept (time 0) and change in HbA_{1c} over time. Models were adjusted for age at death and covariates mentioned above. Continuous variables were centered to the mean of baseline for enhanced interpretation. HbA_{1c} values were logarithmically converted due to the non-normal distribution and then were used in the linear mixed models, so the coefficients can be interpreted as percentage differences in means. We also estimated the difference in HbA_{1c} between survivors and decedents for each year over the 10 years preceding death or follow-up.

Three Cox proportional regression models were used to examine the time-varying association of HbA_{1c} and mortality risk. HbA_{1c} measured in 2006/2008, HbA_{1c} measured in 2010/2012, and HbA_{1c} measured in 2014/2016 were, respectively, the independent variables in the three models. Follow-up was from HbA_{1c} measurements until death or December 2018. Thus the time-varying association of HbA_{1c} with mortality can be tested under different duration of follow-up (as shown in Figure S2). Specifically, HbA_{1c} measured in the three waves were categorized according to the quartiles of HbA_{1c} measurement at baseline. We reported the hazard ratios (HR) and 95% confidence intervals (CIs) for different HbA_{1c} categories in the three models respectively. We choose the third quartile as reference based on the preliminary observations of the

HbA_{1c}-mortality curve and prior studies showing 5.6%-6.5% may be the optimal ranges for overall survival[4]. Proportional hazards assumption was tested by Schoenfeld residuals trend test (all $P > 0.333$). Analyses were adjusted for sociodemographic variables (model 1), additionally for lifestyle variables (model 2), and then for history of hypertension, diabetes and heart diseases, and multimorbidity score (model 3). Restricted cubic splines (RCS) with 4 knots (selected based on model R-square) were applied to visualize the nonlinear association between continuous HbA1c and mortality across different exposure timing.

We performed subgroup analysis by diabetes status at baseline in all of the above analyses. For sensitivity analyses, we additionally adjusted for self-reported medication use relating to hypertension, diabetes, and heart diseases at baseline in all models. Besides, to control for hemoglobinopathy, we excluded participants with HbA1c < 4.5% (n=99). For further sensitivity analyses, we excluded those had a diagnosis of cancer at baseline (n=1701). Finally, we included participants who had missing covariates (n=444) and re-analyzed by imputing the missing values. Statistical significance was set to two-side $P < 0.05$. STATA version 14 and R version 4.1.2 were used for analyses.
